# Ly6G deficiency alters the dynamics of neutrophil recruitment and pathogen capture during *Leishmania major* skin infection

**DOI:** 10.1038/s41598-021-94425-9

**Published:** 2021-07-23

**Authors:** Corinna L. Kleinholz, Monika Riek-Burchardt, Elena A. Seiß, Jonas Amore, Patricia Gintschel, Lars Philipsen, Philippe Bousso, Borna Relja, Burkhart Schraven, Juliane Handschuh, Juliane Mohr, Andreas J. Müller

**Affiliations:** 1 Institute of Molecular and Clinical Immunology, Health Campus Immunology Infectiology and Inflammation (GC-I3), Otto-Von-Guericke-University, Leipziger Strasse 44, 39120 Magdeburg, Germany; 2 Department of Immunology, Dynamics of Immune Responses, Institut Pasteur, 25 Rue du Docteur Roux, 75015 Paris, France; 3 University Clinic for Radiology and Nuclear Medicine, Otto-Von-Guericke-University, Leipziger Strasse 44, 39120 Magdeburg, Germany; 4 Intravital Microscopy of Infection and Immunity, Helmholtz Centre for Infection Research, Inhoffenstrasse 7, 38124 Brunswick, Germany

**Keywords:** Neutrophils, Infection, Inflammation, Innate immune cells, Imaging the immune system

## Abstract

Neutrophils represent one of the first immune cell types recruited to sites of infection, where they can control pathogens by phagocytosis and cytotoxic mechanisms. Intracellular pathogens such as *Leishmania major* can hijack neutrophils to establish an efficient infection. However the dynamic interactions of neutrophils with the pathogen and other cells at the site of the infection are incompletely understood. Here, we have investigated the role of Ly6G, a homolog of the human CD177 protein, which has been shown to interact with cell adhesion molecules, and serves as a bona fide marker for neutrophils in mice. We show that Ly6G deficiency decreases the initial infection rate of neutrophils recruited to the site of infection. Although the uptake of *L. major* by subsequently recruited monocytes was tightly linked with the concomitant uptake of neutrophil material, this process was not altered by Ly6G deficiency of the neutrophils. Instead, we observed by intravital 2-photon microscopy that Ly6G-deficient neutrophils entered the site of infection with delayed initial recruitment kinetics. Thus, we conclude that by promoting neutrophils’ ability to efficiently enter the site of infection, Ly6G contributes to the early engagement of intracellular pathogens by the immune system.

## Introduction

Neutrophils represent the first line of cellular defenses of the immune system during inflammatory responses. Stimulated by recognition of cytokines that are produced upon injury and infection of the tissue, vascular endothelial cells increase surface expression of the adhesion molecules P- and E-selectin^1,2^. Neutrophil surface receptors such as PSGL-1 bind the selectins, which leads to rolling along the endothelium^3^, while chemokine receptors (CR) recognize chemokines expressed by the endothelial cells, which activates their adhesion to the vascular endothelium^4,5^.

The activation of the G-protein-coupled CR leads to a change in the conformation of integrins such as LFA-1 and MAC-1 on the neutrophils^6–8^, eventually stabilizing their adhesion and initiating crawling cell motility^9,10^. The entry of the neutrophils into tissues is mediated by interactions with ICAM-1, ICAM-2, VCAM-1 as well as proteins of cell–cell-junctions such as PECAM-1, CD99, JAM or ESAM^11–14^. MAC-1, which includes the complement receptor 3 (CR3), plays an additional role in recognizing opsonized pathogens and the activation of phagocytes^15,16^.

Once in the inflamed tissue, neutrophils have been shown to be able to adopt a variety of migration patterns to optimally detect the damaged tissue or invaded microbes^17,18^: Their migration to the focus of danger is mediated by gradients of chemoattractants, including danger signals released also during sterile injury and acting directly on the neutrophils^19^, innate immune cell-produced cytokines^20–22^, or pathogen-derived cues^23–26^. In the course of their migration to the target site, neutrophils have been shown to communicate with each other through release of signals such as leukotriene B4 (LTB4) which further enhance their own recruitment and guide them over great distances, resulting in coordinated swarming behavior readily observable by intravital 2-photon microscopy^27^. Such swarm-like behavior has been demonstrated for infections with a variety of pathogens^25,28–31^, but exhibits characteristic features in terms of persistence and number of neutrophils involved for each specific insult^24,25,28,32^.

While integrins are important for endothelial transmigration and invasion into the tissue, it is widely accepted that migration within the interstitial space is largely integrin-independent^33,34^. However, integrin adhesion receptors seem to play an essential role for late phases of neutrophil swarm activity, which are marked by clustering of accumulating neutrophils and remodeling of the extracellular matrix^27^.

Murine neutrophils can be identified via their nearly exclusive expression of Ly6G, a member of the so called Ly6/uPAR family of GPI-anchored surface proteins^35–38^. Only encoded in mice, Ly6G shows structural and functional similarities to another Ly6/uPAR family member, CD177, which is expressed specifically on both human and mouse neutrophils^39^. CD177 interacts with beta-2 integrins as well as PECAM-1, and seems to be involved in migration of neutrophils, since antibody-mediated CD177 blockade can prevent transendothelial extravasation^40–42^. CD177 knock-out animals showed decreased neutrophil numbers in the peripheral blood and the deletion of CD177 further leads to reduced neutrophil accumulation early after infection in a skin infection model with *S. aureus*^43^. Likewise, evidence was found that non-depleting doses of anti-Ly6G antibody can prevent neutrophil entry into the tissue and LTB4-induced neutrophil migration, an effect that was shown to be connected with beta-2 integrin^44^. Furthermore, antibody-mediated ligation of Ly6G was shown to attenuate beta-2 integrin dependent, but not -independent migration of neutrophils into inflamed tissues, underlining that the contribution of beta-2 integrins, and thus the effect of Ly6G ligation to neutrophil recruitment is dependent of the site and stimulus^45–47^. While many of these studies rely on Ly6G ligation via antibodies, genetic Ly6G deficiency, at least in sterile inflammation models in vivo, showed no impact on neutrophil migration^48^. The role of Ly6G deficiency in an infectious setting has, in contrast, not been investigated yet.

In order to elucidate the influence of Ly6G on neutrophil dynamics in an infection model, we here employed cutaneous infection with the intracellular parasite *Leishmania major*. This pathogen exhibits a complex cell tropism: Being taken up mainly by neutrophils upon infection, *L. major* is predominantly found in macrophages and dendritic cell-like monocytes after few days. All these cells, but especially the neutrophils can dramatically change their dynamics when encountering pathogens^25,49,50^. While by numbers, neutrophils are an important host cell mainly in the beginning of the infection, their phagocytosis of and activation by *L.* *major* seems to have an important impact on subsequent induction of adaptive immunity, and thus the whole course of the infection^51–53^. As such, parasite interactions with neutrophils have been shown to activate the immune response^54,55^, but also enable the pathogen to hijack immune activatory functions^56,57^. Furthermore, neutrophils can, by early pathogen sequestration, inhibit the efficient development of an adaptive immune response^58^, but also serve the parasite as a intermediate host cell enabling the infection of further phagocytes by efferocytosis-like mechanisms^59^. Whether Ly6G influences the interactions of neutrophils with *Leishmania* in the course of an infection has remained unknown.

Here, we show that Ly6G deficiency decreases the initial interaction efficiency and pathogen uptake by neutrophils. While later in the infection, we observed slightly decreased numbers of monocyte-derived dendritic cells in Ly6G-deficient animals, the uptake of infected neutrophils by monocytes, pathogen containment and neutrophil speed at the site of infection remained largely unchanged. Intravital 2-photon microscopy revealed that Ly6G-deficient neutrophils entered the site of infection with delayed initial kinetics, underlining the role of this surface protein in mediating interactions with cell adhesion molecules.

## Results

### Ly6G-deficient neutrophils take up less *L. major* during early stages of the infection

**Figure 1:**
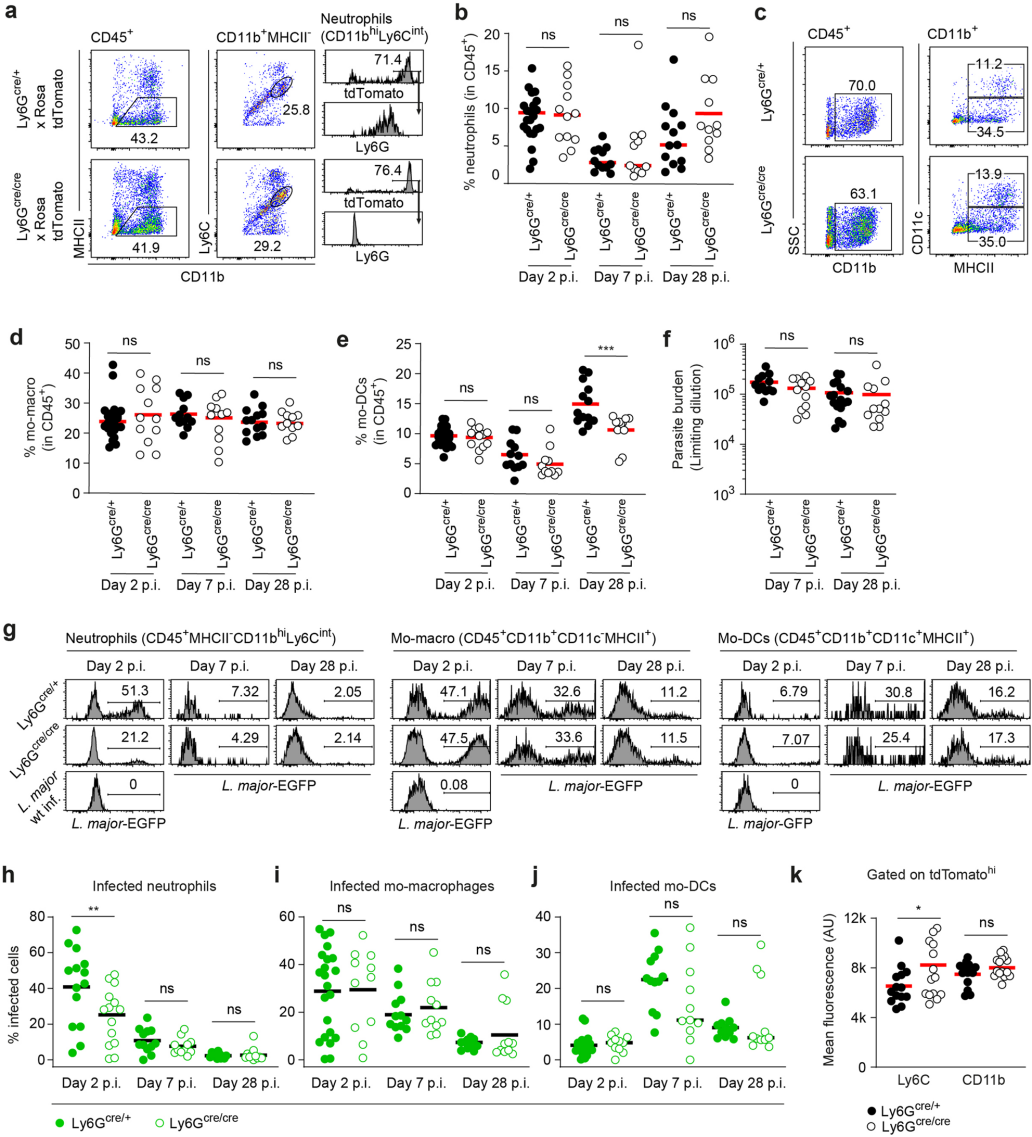
Equal neutrophil recruitment efficiency under Ly6G-deficiency, but decreased uptake of *L. major* parasites during early stage of infection. (**a**) Infection of Ly6G deficient Ly6G^cre/cre^ × Rosa-tdTomato mice and Ly6G proficient Ly6G^cre/+^ × Rosa-tdTomato control mice with EGFP expressing *L. major*. Gating strategy to identify neutrophils lacking the Ly6G marker based on the low MHC class II expression and intermediate expression of Ly6C (CD11b^hi^MHCII^lo^Ly6C^int^) in Ly6G^cre/+^ × Rosa-tdTomato control and Ly6G-deficient Ly6G^cre/cre^ × Rosa-tdTomato mice (left and middle panel). Histogram illustrates the quantification of the tdTomato- and Ly6G-signal on gated neutrophils (CD11b^hi^Ly6C^int^) in Ly6G^cre/+^ × Rosa-tdTomato and Ly6G-deficient Ly6G^cre/cre^ × Rosa-tdTomato mice (right panel). (**b**) Proportion of neutrophils among CD45^+^ cells 2, 7 and 28 days p.i. with 10^6^ metacyclic *L. major*-EGFP parasites in the infected tissue analyzed by flow cytometry. Each dot represents one infected ear. Horizontal bars represent the mean; ns, not significant as determined by two-way ANOVA (time, genotype) with Bonferroni post test for the genotype. (**c**) Recruitment of monocyte-derived phagocytes to the site of infection. Gating strategy for monocyte-derived dendritic cells (moDC, CD45^+^CD11b^+^ CD11c^+^MHCII^+^) and monocyte-derived macrophages (mo-macro, CD45^+^CD11b^+^ CD11c^-^MHCII^+^) in heterozygeous Ly6G^cre/+^ and homozygous Ly6G^cre/cre^ mice. (**d,e**) Percentage of infected (EGFP containing) mo-macro (**d**) and mo-DCs (**e**) among CD45^+^ cells after quantitative evaluation of flow cytometry. Significant decrease of mo-DCs at late infection phase 28 days p.i. Each dot represents one infected ear. Horizontal bars represent the median; ***p < 0.001; *ns* not significant as determined by two-way ANOVA (time, genotype) with pairwise Bonferroni post test. (**f**) Unchanged parasite burden in *L. major* infected ear determined by limiting dilution 7 and 28 days p.i. Horizontal bars represent mean; *ns* not significant as determined by two-way ANOVA (time, genotype) with Bonferroni post test for the genotype. (**g**) Histograms depicting the infection rate of infected neutrophils (left panel), mo-macro (middle panel) and mo-DCs (right panel) after infection with EGFP-expressing *L. major* 2, 7 and 28 days p.i. in Ly6G^cre/+^ and Ly6G^cre/cre^ mice. (**h–j**) Quantitative evaluation of histograms shown in (**g**). Proportion of infected neutrophils (**h**), mo-macro (**i**) and mo-DCs (**j**) analyzed via EGFP fluorescence of the parasite within the cells at day 2, 7 and 28 p.i. in Ly6G^cre/+^ and Ly6G^cre/cre^ mice. Analysis revealed that Ly6G-deficient neutrophils phagocytose less parasites at early infection phase. Each dot represents one infected ear. Horizontal bars represent the mean; **p < 0.01; *ns* not significant as determined by two-way ANOVA (time, genotype) with Bonferroni post test for the genotype. Data are pooled from at least three independent experiments. (**k**) Mean Ly6C and CD11b fluorescence in tdTomato-expressing neutrophils from infected Ly6G^cre/+^ × Rosa-tdTomato and Ly6G^cre/cre^ × Rosa-tdTomato mice. The expression of Ly6C surface protein is increased in Ly6G-deficient animals. Each dot represents one infected ear. Horizontal bars represent mean; *p < 0.05; *ns* not significant as determined by one-way ANOVA with pairwise Bonferroni post test. Data are pooled from at least three independent experiments.

Despite the importance of neutrophils for many aspects of *L.* *major* infection, it is not known how deficiency in the neutrophil-specific Ly6G protein impacts the course of the immune response against this parasite. We therefore set out to infect Ly6G-deficient Ly6G^cre/cre^ × Rosa-tdTomato mice with EGFP-expressing *L.* *major*. Ly6G^cre/+^ × Rosa-tdTomato littermate control mice served as Ly6G-proficient controls. Rosa-tdTomato mice have been shown to exhibit a bright red fluorescence specifically in neutrophils^48^. In order to identify neutrophils lacking the Ly6G marker in the infected ear tissue, we set up a gating strategy based on the low MHC class II expression and intermediate expression of Ly6C for identifying neutrophils independently of Ly6G. Expression of Ly6G-promoter-dependent cre driven from the knock-in alleles in both Ly6G-deficient and heterozygous control animals enabled us to validate the gating strategy (Fig. 1a). Interestingly, between 2 and 28 days of infection, we did not observe any significant differences in neutrophil content in infected tissue (Fig. 1b). As neutrophils have been shown to influence the recruitment of monocyte-derived macrophage- and dendritic cell-like phagocytes, we also determined the fractions of these cells in the infected ear (Fig. 1c). While no difference could be observed between Ly6G-deficient and control animals at early time points of the infection, we detected a small, but significant decrease in the fraction of monocyte-derived CD11c^+^MHCII^+^ cells, namely mo-DCs, at 28 days p.i. (Fig. 1d,e). Pathogen burden remained unchanged both at 1 and 4 weeks p.i. (Fig. 1f).

Analysis of the *Leishmania-*expressed EGFP content of the different phagocyte populations at the site of infection, revealed that at—day 2 post infection, neutrophils in Ly6G-deficient animals had taken up significantly less parasite than cells from control settings (Fig. 1g,h), while the uptake in all other cell types tested was unchanged (Fig. 1g,i,j). Thus, although on the long term perspective, mice lacking Ly6G eventually control *L. major* infection at a comparable efficiency as wild type animals, the Ly6G-deficient neutrophils phagocytose less parasite at early phases of the infection. As Ly6C and CD11b levels have been reported to change with neutrophil activation^60,61^, we determined the mean fluorescence signal of these markers in Ly6G-deficient and control animals. Of note, while we did not observe any difference in CD11b surface expression, Ly6C was (slightly but) significantly increased in Ly6G-deficient animals (Fig. 1k).

### Uptake of neutrophil cellular material by monocyte-derived phagocytes is strictly limited to infected cells, but not impaired in Ly6G-deficient neutrophils

*Leishmania major* infects mainly neutrophils in the first hours of the infection, but are mainly located within macrophage- and dendritic cell-like monocyte-derived phagocytes after a few days of infection^25^. Furthermore, the uptake of neutrophil material by these phagocytes has been shown to occur in the context of their infection with the parasite^59^. Thus, the changed parasite content within neutrophils of Ly6G-deficient mice early after infection might be due to differential cell-to-cell transfer into the monocyte-derived cell types recruited to the site of infection.

**Figure 2:**
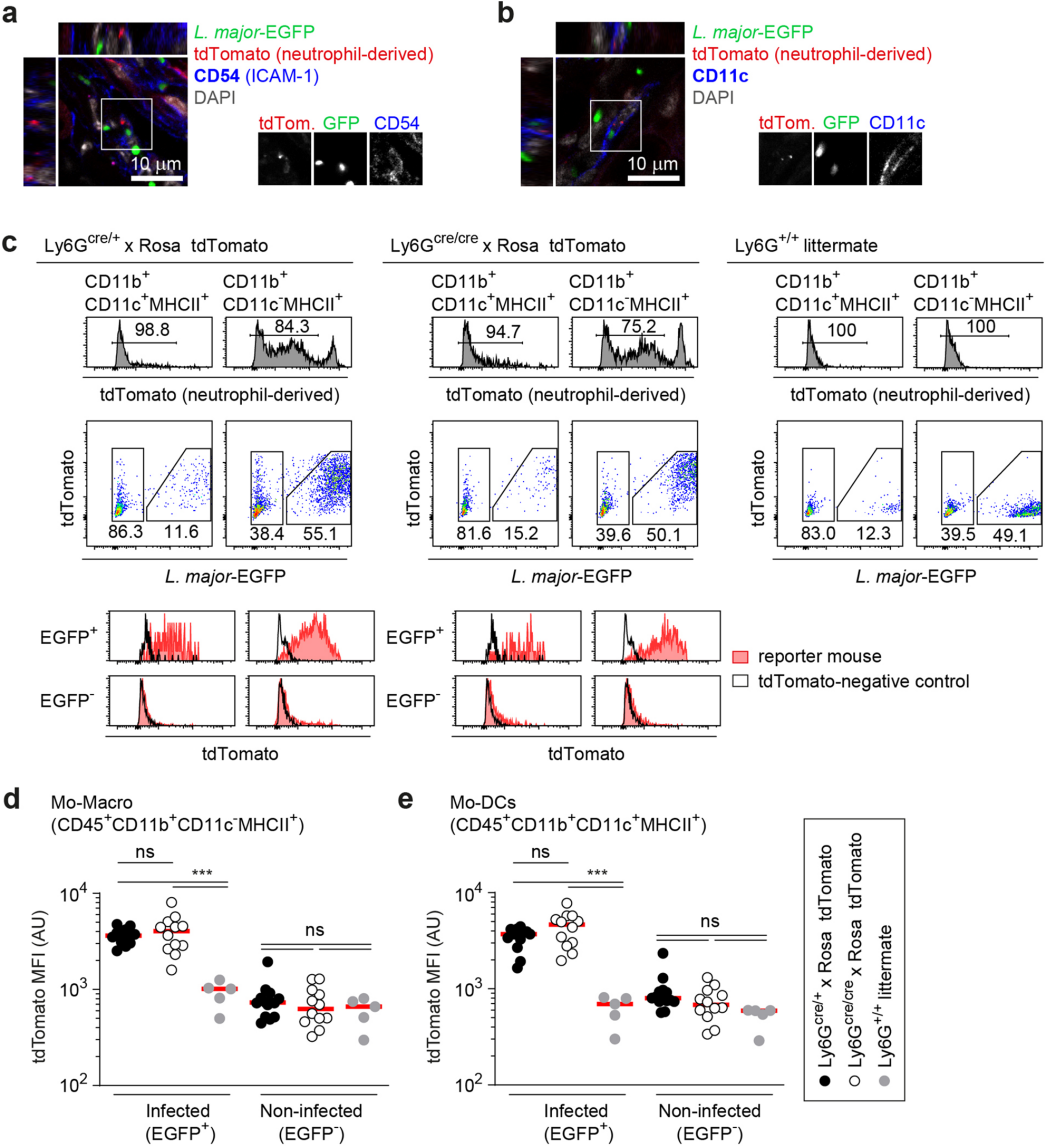
Uptake of neutrophil-derived material by monocyte-derived phagocytes is not altered by Ly6G-deficiency. (**a,b**) Confocal microscopy of Ly6G^cre/+^ × Rosa-tdTomato reporter mice infected with EGFP-expressing *L. major* 2 days p.i., demonstrating concomitant content of *L. major* parasites along with neutrophil-derived material in CD54^+^ (**a**) and CD11c^+^ cells (**b**) and thus suggesting the uptake of *L. major* together with neutrophil material. Parasites are pictured in green and the neutrophil-derived material in red. Scale bar: 10 µm. (**c**) Flow cytometry analysis of infected cells from Ly6G^cre/+^ × Rosa-tdTomato reporter mice (left panel), Ly6G-deficient Ly6G^cre/cre^ × Rosa-tdTomato (middle panel) and non-fluorescent Ly6G^+/+^ littermate control mice (right panel) are depicted. The upper row shows the histograms for quantification of the neutrophil-derived tdTomato signal (omitting the tdTomato^high^ neutrophils) in mo-DCs (CD11b^+^CD11c^+^MHCII^+^) and mo-macro (CD11b^+^CD11c^-^MHCII^+^). Middle row: Gating on infected (EGFP^+^) and non-infected (EGFP^-^) population of phagocytes. Lower row: Exclusive uptake of neutrophil-derived tdTomato material in the *L. major* infected (EGFP^+^), but not the uninfected cell population. Ly6G^+/+^ littermate control mice not expressing tdTomato certify that spectral overlap of EGFP and tdTomato is not responsible for the higher tdTomato-signal in infected cells. (**d,e**) Analysis of the mean fluorescence intensity (MFI) of neutrophil-derived tdTomato signal in control and Ly6G deficient mice for mo-macro (**d**) and mo-DCs (**e**) revealed that tdTomato content is Ly6G independent, Thus, the uptake of neutrophil material by recruited monocyte-derived phagocytes is not altered due to Ly6G deficiency. Infected and non-infected cells are shown from Ly6G^cre/+^ × Rosa-tdTomato (black symbols), Ly6G^cre/cre^ × Rosa-tdTomato mice (white symbols) and Ly6G^+/+^ littermates (grey symbols). Each dot represents one infected ear. Horizontal bars represent mean; ***p < 0.001; *ns* not significant as determined by one-way ANOVA with Bonferroni post test for multiple comparisons.

In order to analyze the concomitant uptake of *L. major* together with neutrophil cellular material, we set out to measure the tdTomato content in monocyte-derived phagocytes of reporter mice expressing the tdTomato in neutrophils. For this, Ly6G-deficient Ly6G^cre/cre^ × Rosa-tdTomato and Ly6G^cre/+^ × Rosa-tdTomato control mice were infected with EGFP-expressing *L.* *major* and analyzed 2 days p.i. Of note, at this time point, we expected efficient uptake of parasites from neutrophils by monocyte-derived cells to have occurred^25^. Confocal microscopy of the site of infection in Ly6G^cre/+^ × Rosa-tdTomato mice revealed that indeed, *L.* *major-*infected cells containing tdTomato material in subcellular compartments could be detected (Fig. 2a), with some of these cells found positive also for the dendritic cell marker CD11c (Fig. 2b). Flow cytometry analysis showed that the uptake of neturophil-derived tdTomato was nearly exclusively limited to infected cells (Fig. 2c). Ly6G^+/+^ littermate controls not expressing tdTomato ensured that any spectral overlap of EGFP with the tdTomato detection was not responsible for the higher signal in infected cells. This underlined that *L. major* transition from neutrophils into different phagocytes early after inoculation occurs together with the uptake of neutrophil material (Fig. 2c–e). However, no significant difference in neutrophil-derived TdTomato content was observable between Ly6G^cre/+^ controls and Ly6G^cre/cre^ animals (Fig. 2d,e). Thus, Ly6G deficiency does not result in altered uptake of neutrophil material by recruited monocyte-derived phagocytes.

### Ly6G-deficient neutrophils enter *L. major*-infected tissues with different kinetics

**Figure 3:**
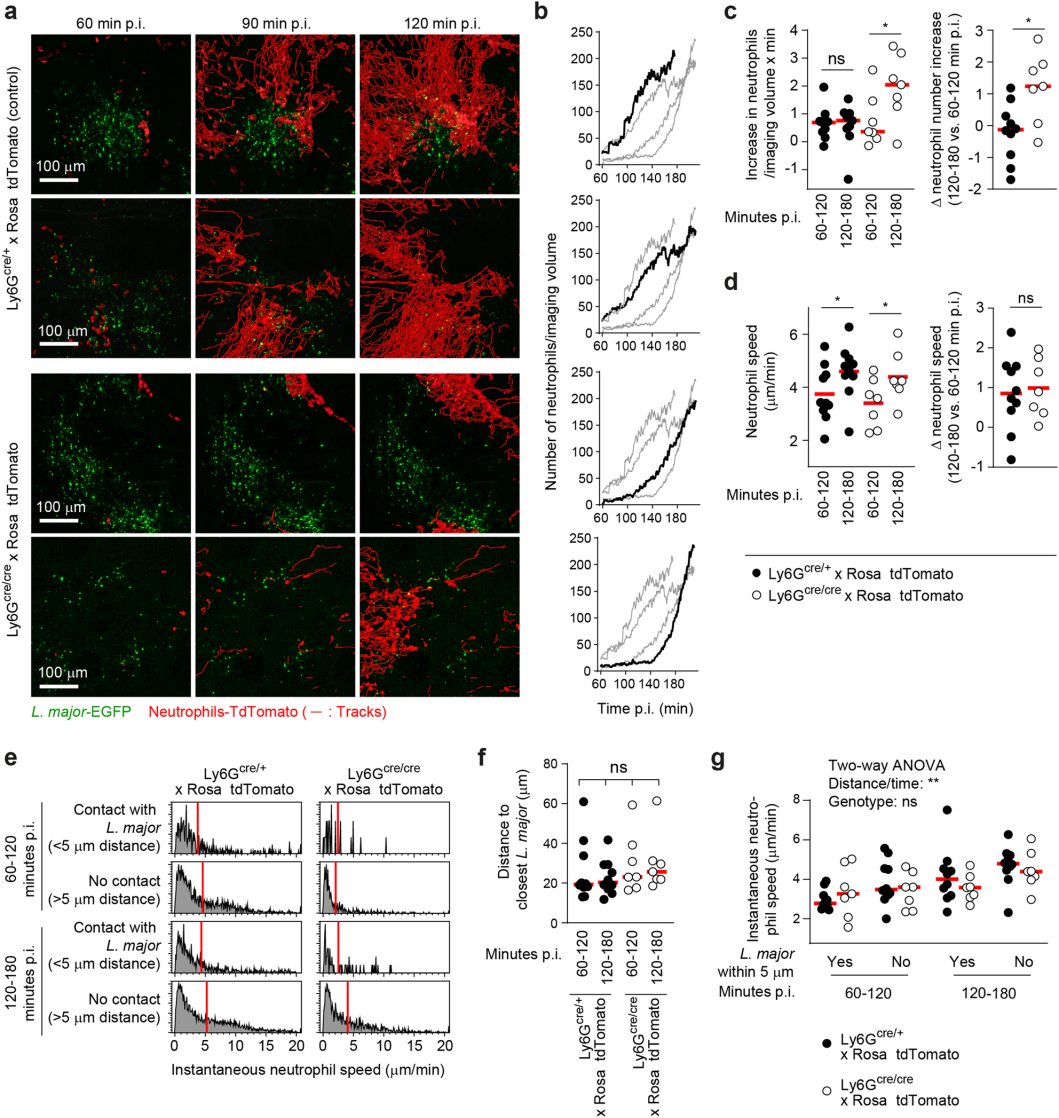
Different kinetics in the early recruitment of Ly6G-deficient neutrophils. (**a**) Intravital 2-photon microscopy of *L. major*-EGFP infected Ly6G^cre/+^ × Rosa-tdTomato (upper panel) and Ly6G^cre/cre^ × Rosa-tdTomato (lower panel) mice 60, 90 and 120 min p.i. shows that neutrophils are recruited to the site of infection within less than 2 h. Red tracks show the accumulated neutrophil migration paths over time. Scale bar 100 µm. (**b**) Numbers of neutrophil shapes present in the imaged volume over time. The curves for the corresponding images in (**a**) are shown in black, the other curves are shown in grey for comparison. (**c**) Left panel: Increase in neutrophil number in the imaged volume per minute, shown separatedly for the time period between 60 and 120, or 120 and 180 min. Data from Ly6G^cre/+^ × Rosa-tdTomato (closed symbols) and Ly6G^cre/cre^ × Rosa-tdTomato (open symbols) is shown. Each symbol represents one infected ear analyzed by intravital 2-photon microscopy. *p > 0.05; *ns* not significant as determined by one-way ANOVA with pairwise Bonferroni post test. Right panel: comparison of the neutrophil increase rate between 60 and 120 versus 120 to 180 min p.i. in Ly6G^cre/+^ × Rosa-tdTomato (closed symbols) and Ly6G^cre/cre^ × Rosa-tdTomato (open symbols) animals. *p < 0.05 as determined by unpaired t test. The initial lower neutrophil recruitment in Ly6Gcre/cre mice was compensated by higher entry rate 2–3 h p.i. (**d**) Left panel: Neutrophil instantaneous speeds, shown separatedly for the time period between 60 and 120, or 120 and 180 min. Data from Ly6G^cre/+^ × Rosa-tdTomato (closed symbols) and Ly6G^cre/cre^ × Rosa-tdTomato (open symbols) is shown. Each symbol represents one infected ear analyzed by intravital 2-photon microscopy. *p > 0.05 as determined by one-way ANOVA with pairwise Bonferroni post test. Right panel: comparison of the neutrophil instantaneous speeds between 60 to 120 versus 120 to 180 min p.i. in Ly6G^cre/+^ × Rosa-tdTomato (closed symbols) and Ly6G^cre/cre^ × Rosa-tdTomato (open symbols) animals. *ns* not significant as determined by unpaired t test. (**e**) Distribution of neutrophil instantaneous speeds in proximity (< 5 µm) and at larger distance from EGFP-expressing *L. major*. Data are shown separately for the time periods between 60 and 120 versus 120 and 180 min p.i. for Ly6G^cre/+^ × Rosa-tdTomato (left panels) and Ly6G^cre/cre^ × Rosa-tdTomato (right panels). (**f**) Analysis of the mean neutrophil distance to EGFP-expressing *L. major*. Data are shown separately for the time periods between 60 and 120 versus 120 and 180 min p.i. for Ly6G^cre/+^ × Rosa-tdTomato (closed symbols) and Ly6G^cre/cre^ × Rosa-tdTomato (open symbols). Horizontal bars denote the median; ns, not significant as determined by one-way ANOVA with Bonferroni post test for multiple comparisons. (**g**) Analysis of neutrophil instantaneous speeds in proximity (< 5 µm) and at larger distance from EGFP-expressing *L. major*. Data are shown separately for the time periods between 60 and 120 versus 120 and 180 min. p.i. for Ly6G^cre/+^ × Rosa-tdTomato (closed symbols) and Ly6G^cre/cre^ × Rosa-tdTomato (open symbols). Horizontal bars denote the median. Data were analyzed using a two-way ANOVA yielding a significant contribution of *L. major* distance and time point of analysis to neutrophil speed, but not of the Ly6G genotype.

Ly6G has been suggested to mediate integrin interaction and extravasation of neutrophils^44–47^. Upon *L.* *major* inoculation, neutrophils are massively recruited within few hours to the site of infection. Thus, we hypothesized that in case the integrin interaction of Ly6G impacts on neutrophil behavior, this would be observable in the kinetics of early recruitment of neutrophils to the parasite. In order to investigate this, Ly6G-deficient Ly6G^cre/cre^ × Rosa-tdTomato and Ly6G^cre/+^ × Rosa-tdTomato control mice were anesthetized, immediately infected with EGFP-expressing *L. major*, and imaged by intravital 2-photon microscopy. As reported previously^25^, neutrophils were recruited to, and accumulating at, the site of infection within less than 2 h, phagocytosing the EGFP-expressing parasites (Fig. 3a). Interestingly, we observed between 1–2 h of infection a substantially higher number of neutrophils entering the site of infection in control mice as compared to Ly6G-deficient mice (Fig. 3a,b). Interestingly, at 2–3 h after infection, the initially lower neutrophil recruitment was compensated by a higher entry rate (Fig. 3b,c). Consequently, while the neutrophil entry rate into the tissue was constant over time in the wild type mice, Ly6G-deficient animals showed a significantly increased neutrophil entry rate between 2 and 3 h versus 1–2 h of infection (Fig. 3c). Of note, the neutrophils accelerated with time in both wild type and control animals, but were unaffected by the Ly6G deficiency (Fig. 3d). Thus, it is conceivable that neutrophil extravasation into the tissue, but not interstitial migration, is influenced by Ly6G.

Neutrophil speed is drastically decreased with the uptake of *L. major* in the tissue^25^. In order to investigate the initial interaction of neutrophils with the parasite, we sought to quantify differential changes in cellular speeds linked to the distance between *L.* *major* and recruited neutrophils. For this, we compared the instantaneous speed of neutrophils in close proximity (less than 5 µm) or not contacting *Leishmania* (more than 5 µm) in wild type and Ly6G-deficient settings (Fig. 3e). As shown in Fig. 3f,g, neutrophil instantaneous speed was affected both by distance from *Leishmania* and time point of infection, but not by a lack of Ly6G. Therefore, although the recruitment of neutrophils lacking Ly6G is delayed early after infection with *L.* *major*, the neutrophil speed within the infected tissue is not affected.

## Discussion

Ly6G is among the most important markers used to identify neutrophils in mice^48,62^, however whether it has a function in enabling neutrophils to be recruited to and engage pathogens during infection is unclear. Although Ly6G is present in mice, but not humans, elucidating its role for the dynamics of immune responses might reveal important differences between mouse models and human diseases, enabling the refinement of preclinical experimentation in order to more closely recapitulate clinical settings.

To study the role of Ly6G deficiency in neutrophils, we have employed the well-characterized model of *L.* *major* infection in a C57BL/6 mouse background, which is characterized to control the parasite efficiently^63–65^. Also, the initial dynamics of neutrophils and early host cell tropism of *L. major* at the site of infection has been intensely studied in this system^25,56,59^. This is relevant since the role of neutrophils while crucial cells for early defence against infections was shown to depend on the combination of *Leishmania* species and mouse model used^53,66,67^.

Antibody-mediated ligation of Ly6G attenuates beta-2 integrin dependent migration of neutrophils^44^. Furthermore, being a constituent of the MAC-1 integrin, CR3 has been shown to impact on phagocyte activation as well as the interaction with and uptake of pathogens^15,16,68,69^. With respect to the evidence for a crosstalk between Ly6G and integrin activation we hypothesized that the early interaction of immune cells, in particular neutrophils, with *L.* *major* might be influenced by Ly6G deficiency. Indeed, analysis of the uptake of EGFP-expressing *Leishmania* into neutrophils revealed that the early uptake of the parasite is decreased in Ly6G-deficient animals, although parasite was found to be unchanged between wildtype and Ly6G-deficient animals eventually. The finding that as late as 4 weeks p.i., we find slight changes in recruited monocyte-derived dendritic cell content in the infected tissue underlines that early changes in neutrophil interaction with the parasite can affect the induction and execution of the adaptive immune response, as shown previously^58,70^. The finding that we do not observe differences in pathogen burden, however, indicates that these changes are compensated by other containment mechanisms, as has been shown for other immunodeficiencies during *Leishmania* infection^71–73^.

Neutrophils have the potential to kill phagocytosed intracellular pathogens such as *Leishmania*. However, these immune cells can also serve as the first host cells for disease-transmitting parasites, with the intracellular killing mechanisms being evaded by the pathogen. Parasitized neutrophils were suggested to function as a ‘Trojan horse’, to transfer *Leishmania* silently to macrophages^25,74,75^. In vivo imaging has contributed a second evasion mechanism called ‘Trojan rabbit’ strategy, whereby parasites escape apoptotic neutrophils to infect macrophages^76,77^. According to this notion, the dying neutrophil silences host macrophages to enable a productive *Leishmania* infection. Moreover, the parasite induces neutrophil apoptosis attracting macrophages and dendritic cells, and finally encourages engulfment of the parasite containing neutrophils by these terminal host cell preferred by *Leishmania*^25,59^. It is also important to mention that it was recently shown that efferocytosis itself is important for subsequent responses by the adaptive immune systems^78^.

Using Ly6G reporter mice in which neutrophil cellular material can be tracked by tdTomato fluorescence, we show that the uptake of neutrophil material by monocyte-derived macrophages and dendritic cells was only efficient in infected cells. Two explanations are conceivable: either *L. major* itself forces the concomitant uptake of neutrophils by these cells very strongly, or the parasite inhibits the degradation of neutrophil material in infected as compared to uninfected phagocytes. While Ly6G does not influence the uptake by phagocytes, the possibility of an efferocytosis forced by *Leishmania* might be an important concept to be investigated in future studies.

Using intravital 2-photon microscopy, we found no change in neutrophil speed, but a significant delay in tissue entry for Ly6G-deficient neutrophils as compared to wildtype. These different entry kinetics might also be linked with the slight but significant increase in Ly6C, which has been reported to change rapidly with neutrophil^60^, but not monocyte activation. While the lack of drastic differences in interstitial motility underlines the findings of intergrin independency during this stage^33,34^, we observe a compensation of the initially lower neutrophil entry rate by an increased entry rate 2–3 h p.i. Thus, if the inflammatory signals from the site of infection become sufficiently strong, this might result in efficient neutrophil extravasation also in a Ly6G-deficient setting. The decreased proportion of infected neutrophils at early time points p.i. observed in Ly6G-deficient animals could result from the inefficient initial tissue entry of these cells, which is likely to result from the altered integrin interactions^45^.

Besides the reported impact on integrin activation, recent studies suggest further, so far unrecognized functions of Ly6G: For example, single cell RNA sequencing revealed that Ly6G ligation might not, as previously suggested, mainly result in depletion of the neutrophils, but in shifts in neutrophil populations with respect to circadian rhythm-related neutrophil turnover^79,80^. Our observation that Ly6G deficiency impaired early neutrophil engagement of pathogens through delayed entry to the site of infection, therefore also opens interesting questions on the role of different neutrophil subpopulations in the control of intracellular pathogens.

## Materials and methods

### Pathogen

EGFP-expressing parasites (kindly provided by T. Aebischer, Robert Koch Institute, Berlin, Germany) were generated by stable integration of the EGFP coding sequence^81^ into a small subunit (18S) rRNA-coding locus of *L. major* LRC-L137 V121^82^. Parasites were grown in M119 medium supplemented with 10% heat-inactivated fetal calf serum, 10 mM adenine, 1 mg/ml biotin, 5 mg/ml hemin, 2 mg/ml biopterin (all from Sigma) and 7.5% (m/v) NaHCO_3_ at 26 °C for a maximum of 6 passages. Maintenance of EGFP transgene in transgenic strains is associated with co-inserted hygromycin B resistance gene, hence hygromycin B was routinely added to parasite culture.

### Mice

C57BL/6J mice were bred in the Central Animal Laboratory (ZTL) of the Medical Faculty of Otto-von-Guericke-University Magdeburg, or purchased from Charles River (Sulzfeld, Germany). The C57BL/6-*Ly6g*^*tm2621 (Cre-tdTomato) Arte*^ mice^48^ were bred in the ZTL. For efficient tracking of neutrophil material via tdTomato fluorescence, these mice were crossed with B6.Cg-*Gt(ROSA)26Sor*^*tm14(CAG-tdTomato)Hze*^ for enhanced tdTomato fluorescence.

All mice were housed under SPF conditions, sex- and age-matched animals between 8 and 14 weeks were used for infections. Littermate controls were used in all experiments. All procedures involving animal experimentation were reviewed and approved by the Ethics Committee of the Office for Veterinary Affairs of the State of Saxony-Anhalt, Germany (permit license numbers IMKI/G/03-1253/14, IMKI/G/01-1314/15 and IMKI/G/01-1575/19) in accordance with legislation of both the European Union (Council Directive 499 2010/63/EU) and the Federal Republic of Germany (according to § 8, Section 1 TierSchG, and TierSchVersV). All animal work was performed and reported according to the ARRIVE guidelines.

### Infection experiments

For high dose infection of mice (flow cytometry experiments), stationary phase cultures were centrifuged (1000×*g*, 10 min, RT) and resuspended in PBS. 10^6^ Parasites were subsequently injected into the ear dermis. For low dose infection (intravital 2-photon microscopy experiments), metacyclic parasites were purified from stationary phase cultures via incubation with 50 µg/ml unconjugated peanut agglutinin (Vector Laboratories, #L-1070) in a volume of 1 ml *Leishmania* culture medium at room temperature for 20 min. Agglutinated non-metacyclic parasites were removed by centrifugation at 100×*g* for 10 min at room temperature. The supernatant was carefully transferred to a 15 ml Falcon tube, which was filled up with *Leishmania* culture medium and again centrifuged at 1000×*g* for 10 min at RT. After discarding the supernatant, the pellet containing the parasites was subsequently taken up in fresh, 4 °C cold PBS (Dulbecco without Calcium and Magnesium, L 1820), spun down at 1000×*g* for 5 min at RT and taken up in PBS for a final concentration of 2 × 10^4^ *L. major* per µl, of which 0.5 µl were injected into the ear dermis.

### Cell isolation

Infected ears harvested from euthanized mice were separated into dorsal and ventral sheets using tissue forceps before being digested for 60 min at 37 °C while shaking at 600 rpm in RPMI medium 1640 supplemented with 100 U/ml Penicillin/Streptomycin (Gibco), 0.5 mg/ml liberase TL (Roche) and 50 µg/ml DNase (Invitrogen). Single cell suspensions in PBS were prepared by crushing digested ears through a 70 µm cell strainer and employed for limiting dilution assay or flow cytometry. For better purity, cells were MACS-isolated using anti-CD45 microbeads (clone 30F11.1, Miltenyi, Catalog 130-052-301) according to manufacturer’s instructions before staining and analysis by flow cytometry.

### Flow cytometry

Samples were Fc-blocked using anti-CD16/32 antibody (TruStainFcX, clone 93, BioLegend, 10 µg/ml, Catalog # 101320) before fluorescent antibody staining with Ly6G-Brilliant Violet 421 (clone 1A8, Biolegend, 0.5 µg/ml, Catalog # 127601), CD45.2-PerCP/Cy5.5 (clone104, Biolegend, 0.5 µg/ml, Catalog # 109827), Ly6C-PE/Cy7 (Clone HK 1.4, Biolegend, 2 µg/ml, Catalog # 128017), CD11b-APC/Cy7 (clone M1/70, Biolegend, 0.5 µg/ml, Catalog # 101225) and CD11c-APC (clone N418, Biolegend, 1 µg/ml, Catalog # 11730925). Analysis was performed with Fortessa or FACSAria (BD Biosciences) flow cytometers/sorters equipped with excitation lasers at 405 nm, 488 nm, 560 nm, and 633 nm. Data were analyzed by using the FlowJo 10.6.2 software (FlowJo, LLC).

### Limiting dilution assay

Quadruplicate samples of each homogenate were serially diluted in 1:2 steps in *Leishmania* culture medium. The initial parasite number was determined from the median of the triplicates after 14 days at 26 °C from the highest dilution which parasite exhibited growth.

### Immunofluorescence microscopy

Three days after infection, ears were fixed in 1 ml of 4% PFA for 2 h, placed in 20% sucrose-PBS solution and transferred into Tissue-Tec O.C.T. medium, followed by snap freezing with liquid nitrogen. 20 µm thick cross-sections were prepared, drawn on Superfrost slides and then dried for 2 h at RT. The staining steps were carried out at RT. Slides were permeabilized with 0.2% Triton X-100 for 10 min and blocked with 10% BSA for 1 h, before staining with primary antibody (Armenian Hamster anti-CD11c (clone HL3, BD Biosciences, 1.25 µg/ml, Catalog # 550283) or Armenian Hamster anti-CD54 (clone 3E2, BD Biosciences, 1.25 µg/ml, Catalog # 550287) and AF647-marked polyclonal anti-Armenian Hamster secondary antibody (Jackson ImmunoResearch, 7 µg/ml, Catalog # 127-605-160) for 1 h incubation time each. For nuclear staining DAPI (Sigma-Aldrich, 1 µg/ml, 10 min) was used. Samples were analyzed with a confocal microscope TCS SP8 (Leica Microsystems) at × 40 magnification with a HC PL APO CS2 40×/1.30 OIL objective. Lasers with wavelengths of 405 nm, 488 nm, 561 nm and 633 nm were used for excitation. Image inspection, superimposition and cropping was performed using the ImageJ software (http://imageJ.nij.gov/ij).

### Intravital two-photon imaging

The mice were injected with 100 mg/kg Ketamin and 10 mg/kg Xylazin i.p. and supplemented with 3 mg/kg Acepromazin i.p. after entering anaesthesia. The ventral side of the ear was prepared for intravital 2-photon microscopy as described previously^83,84^. In brief, the animals were placed on a heating stage adjusted to 37 °C and the ear was fixed to a metal platform using double-sided tape and covered with Vidisic carbomer gel (Bausch + Lomb). A coverslip sealed to a surrounding parafilm blanket was placed onto the ear and fixed above the ear. Intravital 2-photon imaging was performed using Zeiss LSM 700 upright microscope equipped with a Mai Tai DeepSee Ti:Sa laser (Spectra-Physics) tuned to 980 nm. The emitted signal was sequentially split by 625 nm (reflected), 490 nm and 560 nm long pass dichroic mirrors and filtered with 485 nm SP (second harmonics), 525/50 nm (EGFP), 589–654 nm (tdTomato), filters before collection with non-descanned detectors.

The analysis was carried out using the Imaris analysis software using the Isosurface tool. Neutrophils could be marked and assigned to a position with x, y and z coordinates using the tracking function. Calculation and analysis of neutrophil speeds and distances to *L.* *major* was performed from datasets extracted from the Imaris software using ImageJ (http://imageJ.nij.gov/ij) and the DISCit software^85^.

### Statistical analysis

One-way analysis of variance (ANOVA) with appropriate multiple comparison post-tests (indicated in the respective figure legends) was employed to compare multiple samples or pairwise analysis within datasets with more than two experimental groups. For time course experiments, two-way ANOVA with genotype and time point of infection was employed as indicated in the figure legends. Two-group comparisons were made by two-sided, unpaired t tests. Data are always presented as mean with each individual sample. Statistical analysis, including Shapiro–Wilk normality testing of the acquired data, was performed using the Prism software (Version 8.0, GraphPad).

## Data Availability

The raw data that support the findings of this study are available from the corresponding author upon request.
